# Supplementary material for: Exploring the Intersection of Microplastics and Black Soldier Fly Larvae: A Comprehensive Review
Source: Insects. 2025 Sep 1;16(9):913. doi: 10.3390/insects16090913 (PMC12470764; doi:10.3390/insects16090913)
Supplement: Supplementary file 1 [file insects-16-00913-s001.zip › insects-3831967-supplementary.pdf]

File S1

PRISMA flow diagram for new systematic reviews which included searches of databases only (16.08)

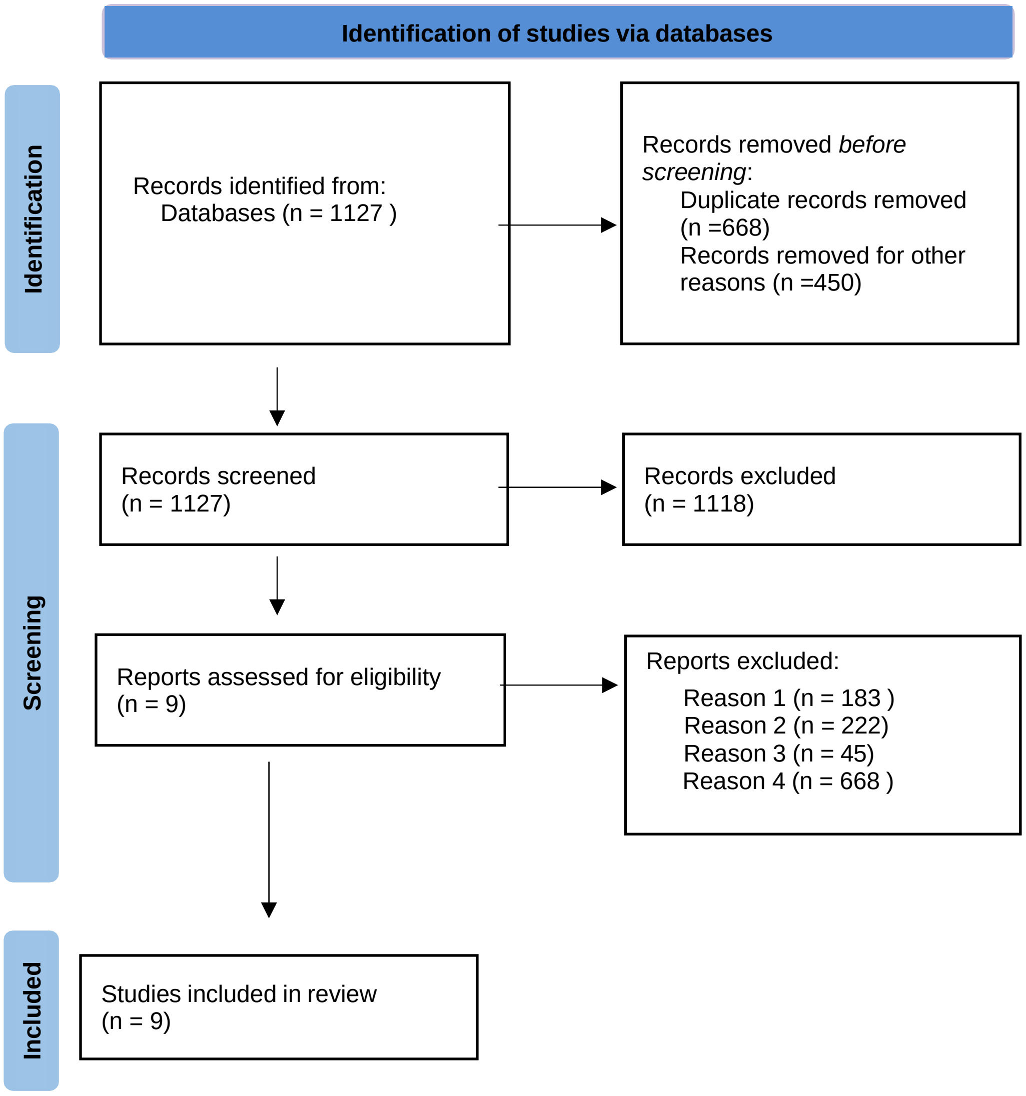

Reason 1 – "Black soldier fly larvae"/"Hermetia illucens" is not found in the content

Reason 2- "Microplastic" and various types of

microplastics are not found in the content

Reason 3- none of the following "Black soldier fly larvae", "Microplastic" and various types of

microplastics are found in the content

Reason 4- duplicate
